# Congenital Hypothyroidism: Moving Ahead, but a Long Way Still to Go

**DOI:** 10.3390/ijns11040093

**Published:** 2025-10-13

**Authors:** Ernest M. Post, Natasha L. Heather

**Affiliations:** 1 Cooper Medical School of Rowan University, Camden, NJ 08103, USA; 2 New Zealand Newborn Metabolic Screening Programme, LabPlus, Health New Zealand|Te Whatu Ora, Auckland 1023, New Zealand; nheather@adhb.govt.nz; 3 Liggins Institute, University of Auckland, Auckland 1023, New Zealand

Newborn screening (NBS) for congenital hypothyroidism (CH) has been going on for more than fifty years, but we are still learning more about the process and the disease(s). This Special Issue of the *International Journal of Neonatal Screening* was conceived to focus on key contemporary issues surrounding CH NBS. These key issues include testing algorithms, CH NBS in low- and middle-income countries, premature infants, central hypothyroidism, and uncommon causes of CH.

We are very pleased to have met most of those goals. This Special Issue includes ten articles from Algeria, Australia, Brazil, Canada, Chile, China, Indonesia, Italy, Morocco, and New Zealand. The breadth of the information ranges from a report on the effects of not having CH NBS (Contribution 1) (lest we forget) to advocating for the expansion of CH NBS to include very rare causes (Contribution 2). The papers demonstrate both how far we have come (99.5% of newborns screened in one jurisdiction) and how much more we still have to accomplish (two thirds of patients experienced hypothyroidism after having been made euthyroid) (Contribution 3). 

The papers included in this Special Issue touch on the high points of the advancements needed in CH NBS. While not explicitly part of the Special Issue, the most important goal is to increase universal CH NBS from, at present, approximately 30% worldwide coverage to 100% [1] (Contributions 4 and 5). Other major issues include providing good care to those with CH (Contribution 3), reviewing screening cutoffs (Contribution 6), finding the best protocol for re-screening premature babies (Contributions 7 and 8), identifying the causes of the rise in CH (Contribution 9), and discovering the best use of genetic information in CH NBS (Contribution 10).

We look forward to future publications on the efforts undertaken to address these areas.
